# Supplementary material for: Effect of silver diamine fluoride and proanthocyanidin on resistance of carious dentin to acid challenges
Source: PLoS One. 2020 Sep 17;15(9):e0238590. doi: 10.1371/journal.pone.0238590 (PMC7498094; doi:10.1371/journal.pone.0238590)
Supplement: Attachment — Submitted filename: Comments for PONE-D-20-09551.docx [file pone.0238590.s003.docx]

The work by Fateme et al. demonstrated that the mechanical properties of dental caries could be significant improvement by using Silver diamine fluoride (SDF) modification. It also investigated the microstructure of the dentin after acid challenges. However, the authors have not deeply researched the mechanism of the SDF or GSE on how to protect the dentin against acid. Meanwhile, many statements and conclusions the authors raised have no direct evidence to support. In a word, this work is suitable for publication in PLOS ONE, but need to provide more experimental data as evident and addressing the following concerns:

1. Missing a unit after “0.5 ± 0.1”, please see Line 123.

2. Does there exist an interaction between dentin and PA from GSE or SDF? If YES, what is the interaction between them? You can examine it through spectroscopic technology such as FTIR.

3. What is the cubic particles shown in Fig 4, which is important to understand the role of GSE and SDF?

4. The authors think that the SDF treatment can improve the mechanic properties of carious dentin and attributed it to increased mineral content. Here the authors just provide SEM images, which only qualitative analysis, and no other direct evidence such as TEM image. For TEM image can refer the following references.

*[1] Wang, J. et al. Remineralization of dentin collagen by meta-stabilized amorphous calcium phosphate. CrystEngComm* ***15****, 6151-6158, doi:10.1039/C3CE40449H (2013).*

*[2] Sun, J. et al. Biomimetic promotion of dentin remineralization using l-glutamic acid: inspiration from biomineralization proteins. J. Mater. Chem. B 2, 4544-4553, doi:10.1039/C4TB00451E (2014).*

5. The reaction between hydroxyapatite and SDF is inaccurate, which included the formula of hydroxyapatite (Ca_10_(PO_4_)_6_(OH)_2_) and the stoichiometric numbers. Please correct the reaction, See Line 258.

6. The sentence *“It has been proposed that PA can bind to metallic ions via some functional groups.”* should be added a reference to support it.

7. The style of some of the list of references is incorrect, like reference #15, #16 and so on. Please according to the journal criterion to correct it.
