# Supplementary material for: Effect of silver diamine fluoride and proanthocyanidin on resistance of carious dentin to acid challenges
Source: PLoS One. 2020 Sep 17;15(9):e0238590. doi: 10.1371/journal.pone.0238590 (PMC7498094; doi:10.1371/journal.pone.0238590)
Supplement: Attachment — Submitted filename: PONE-D-20-09551_reviewer.docx [file pone.0238590.s004.docx]

This manuscript addresses inhibition of carious dentin via silver diamine fluoride and proanthocyanidin. The study has some strength including different methods to verify the treatment effect, but major concerns exist. See the below points to address.

- Page 6 line 134: In your experiment, specimens were treated 30 mins in GSE groups, however, specimens were treated 4 mins in SDF groups. It not clear to me why the treatment time is different? Please rectify.
- Page 10 line 205: The statement “bacterial cell bodies were present” in fig 1a, 1b. I actually not looking at the bacterial cell bodies, just the dentine in the SEM. Please verify and make an obvious mark.
- Page 10 line 208: It is unclear based on the SEM shown that resistant capacity to demineralization was assessed. I think SEM only used to observe the surface demineralization of carious dentin. However, this study requires additional experiments to confirm your conclusion. For example, observation of internal demineralization of dentin slices using TEM.
- Fig2a: Why the magnification of SEM images in Fig2a (3x) is different from Fig1a, Fig3a and Fig4a (5x)? Please provide your rationale for your choice.
- All SEM figures: Please provide more statistical analysis of resistant capacity to demineralization such as the amount of granular deposit between control, GSE group, SDF group and GSE+SDF group.
